# The Neuroscience of Nonpharmacological Traditional Chinese Therapy (NTCT) for Major Depressive Disorder: A Systematic Review and Meta-Analysis

**DOI:** 10.1155/2019/2183403

**Published:** 2019-05-15

**Authors:** Jiajia Ye, Wai Ming Cheung, Hector Wing Hong Tsang

**Affiliations:** ^1^Department of Rehabilitation Sciences, The Hong Kong Polytechnic University, Hunghom, Hong Kong; ^2^Faculty of Education, The University of Hong Kong, Pokfulam, Hong Kong

## Abstract

**Background:**

Depression is a common disease affecting a large number of people across the world. Many researchers have focused on treatment for depression based on Western scientific approaches, but research based on traditional Chinese medicine (TCM) interventions, studying its clinical effectiveness and the underlying mechanisms involved, has been limited. The aim of this review is to conduct a pioneering systematic review with meta-analysis of existing studies that investigate the neuroscience basis of nonpharmacological traditional Chinese therapy (NTCT).

**Methods:**

Both English (*Pubmed*,* Embase*,* Scopus*,* SPORTDiscus*,* PsycINFO*) and Chinese (*China National Knowledge Infrastructure* (*CNKI*)) databases were searched from inception to October 2018. The effects of NTCT on major depressive disorder, brain activity, and neurophysiological biomarker related outcomes were extracted. Study quality was assessed using the Physiotherapy Evidence Database (PEDro) scale. The effect size of each study was reported by the mean difference of change scores.

**Results:**

Six of twelve eligible studies showed that there was a significant improvement in favor of acupuncture in depressive symptoms (SMD -0.69, 95%* CI* -1.09 to -0.28,* p*=0.002, *I*^2^ = 73%,* p*< 0.0008). Based on the available evidence, NTCT including acupuncture, Qigong, and Tai Chi was found to possibly improve brain metabolites, brain activity, and immune and endocrine systems in patients with major depressive disorder.

**Conclusions:**

Acupuncture could effectively relieve depressive syndromes. The clinical effects of acupuncture might be attributable to their influence on three proposed pathways, namely, the hypothalamic–pituitary–adrenal (HPA) axis, the locus coeruleus (LC)-immunity pathway, and the negative feedback loop of the hippocampus. Nevertheless, conclusions are limited due to the small number of studies included and the low-quality of the study designs. In the future, a cross-sectional study is needed to test the proposed plausible pathways. PROSPERO registration number is CRD42017080937.

## 1. Introduction

Major depressive disorder (MDD) is a common but serious mental illness. The prevalent fast and furious life and work rhythm is associated with an increase in this mental illness, and it is projected to be the second greatest cause of disability worldwide by 2020 [1, 2]. Furthermore, it affects more than 350 million people, especially those who reside in industrialized and urban areas. The incidence rate of MDD is around 6% in the general population [3], with a lifetime percentage reaching 16% [4, 5]. Symptoms of MDD vary from fatigue, depressed mood, emotional disturbance, poor appetite, sleeping problems, cognitive impairment, and feelings of worthlessness or excessive guilt to suicidal thoughts. Individuals with MDD who experience these symptoms have a reduced quality of life; in addition, it places a heavy economic burden on their families and society [6]. Thus, MDD has currently been targeted as one of the most serious health issues faced by industrialized societies and requiring more urgent attention.

In the past two decades, antidepressant medication (e.g., selective serotonin reuptake inhibitors (SSRIs)) has been used for MDD as a mainstream treatment [7]. Significant effects on reducing levels of depression are widely accepted, but these pharmacological treatments cause negative results (e.g., nausea and vomiting), particularly in long-term users [7]. Beyond this medication, cognitive behavioral therapy (CBT) and counseling are alternative methods for treating MDD [8]. It is worth noting that these nonpharmacological treatments are costly, time-intensive, and not suitable for all individuals with MDD [9–11]. Given this fact, it is necessary to explore other methods for treating MDD like nonpharmacological traditional Chinese therapy (NTCT).

NTCT originated in ancient China and has a long history spanning thousands of years [12]. Mind-body therapies like Tai Chi, Qigong, and acupuncture are core parts of NTCT, which emphasize the integration of mind (brain) and body in practice. In recent years, there has been an increasing worldwide interest in the clinical application of NTCT treating depression, and beneficial effects have been observed in some studies [13, 14]. Beyond this, imaging studies have investigated the potential neuromechanism of beneficial effects of NTCT for MDD [15, 16]. To date, no systematic review has been undertaken to synthesize the existing literature on this topic. Thus, the aim of this review is to summarize the neural basis for the clinical evidence of the effectiveness of NTCT in patients with MDD and to gain a better understanding of the pathological pathways of depression based on NTCT.

## 2. Methods

This systematic review has been registered on the international prospective register of systematic reviews by the authors (PROSPERO registration number CRD42017080937).

### 2.1. Information Sources

This systematic review with meta-analysis has been conducted in accordance with the preferred reporting items for systematic reviews and meta-analyses (PRISMA) guidelines. Two independent reviewers (Ye and Cheung) served as the systematic reviewers of the following databases:* Pubmed, Embase, China National Knowledge Infrastructure (CNKI), SPORTDiscus, Scopus, *and* PsycINFO*, from their inception to October 2018. The following main keywords were used in the systematic search: depression, major depressive disorder, affective disorder, neuroscience, neuroimag*∗*, neurotransmitter, complementary therapies, traditional Chinese, mind-body therapies, and Qigong (see the appendix). The reference sections of relevant articles were also reviewed by the authors. Languages were restricted to English and Chinese.

### 2.2. Study Selection and Eligibility Criteria

The titles and abstracts of all the articles obtained through the search were independently screened by the two researchers. Studies were included if they (1) focused on a kind of NTCT (e.g., acupuncture, acupressure, auricular therapy, massage, Qigong, moxibustion, and Tai Chi); (2) involved patients diagnosed as MDD based on any valid and clinical diagnostic criteria [17]; (3) recruited adults (aged above 18); (4) had a control group; (5) targeted outcomes using depression scales with at least one neuroscience measurement including electroencephalography (EEG), functional magnetic resonance imaging (fMRI), magnetic resonance imaging (MRI), positron emission tomography (PET), single-photon emission computed tomography (SPECT), and functional near-infrared spectroscopy (fNIRS) but were not limited to biomarkers such as blood, saliva, and urine samples; (6) were full papers written in English or Chinese; and (7) were published in peer-reviewed journals. Studies were excluded if (1) the full text was not available; (2) they had a focus on children (under the age of 18); (3) they had a focus on pharmacological treatments; (4) they used outcome measures without depression scales and neuroscience assessments; and (5) they were literature or systematic reviews. Any disagreement between the reviewers was resolved by discussion under the supervision of the corresponding author who is an experienced and seasoned researcher in integrative medicine.

### 2.3. Data Extraction and Management

Data extraction from selected studies included the characteristics of the population, diagnoses, interventions, study design, and outcomes. Consensus was reached via a discussion if there were disagreements between the two reviewers.

### 2.4. Study Quality Assessment

The 11-item PEDro scale was used to measure the methodological quality of the clinical studies [18, 19]. It involved eligibility criteria, randomization, concealed allocation, similar baseline, blinding of participants, blinding of therapists, blinding of assessors, key outcome measures from more than 85% of the subjects, intention-to-treat, between group difference, and both point measures and measures of variability. The maximum total score was ten because the first item (eligibility criteria) did not contribute to the total score. Points were awarded when a criterion was clearly satisfied [20].

### 2.5. Data Analysis

Meta-analysis was conducted through Review Manager (version 5.3, the Nordic Cochrane Centre, Copenhagen, Denmark) on depressive outcomes. The mean changes of the depressive outcomes between the baseline and posttreatment measurements were computed for both the experimental and control groups in eligible studies. The effect size of each study was reported by the mean differences of change scores. When the 95% confidence interval (CI) did not include 0 or the* p*-value was less than 0.05, the* CI* and statistical significance were reported. The standardized weighted mean difference (*SMD*) method was performed to obtain the pooled estimates of effect size for studies that reported the same outcome by different scales. The homogeneity of included studies was assessed by the Chi-squared (*I*^2^) test. The publication bias was assessed by funnel plot, along with the Egger's Regression Test.

## 3. Results

### 3.1. Search Selection

**Figure 1:**
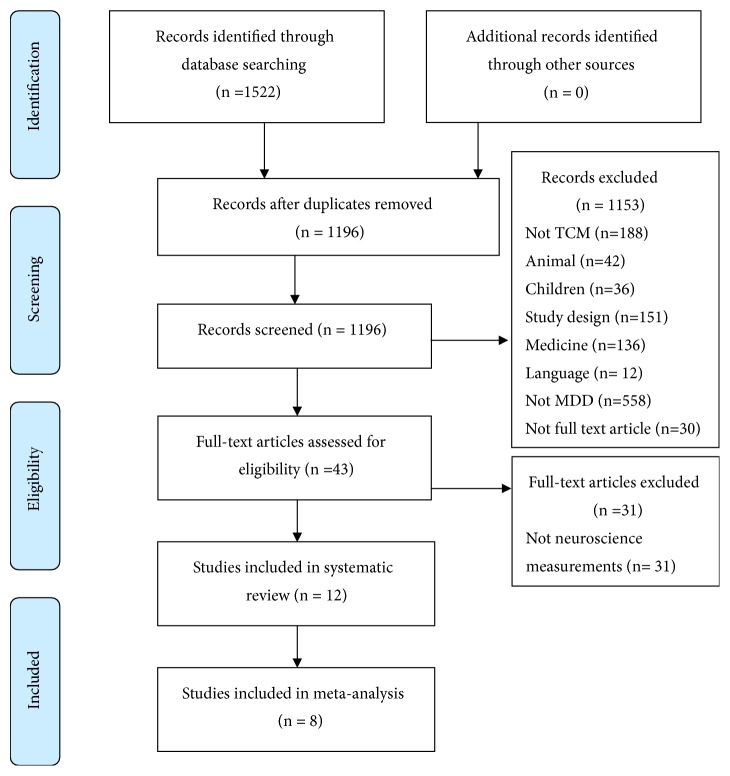
PRISMA flow chart of study selection process.

Both electronic and manual searches resulted in 1522 records in total. Forty-three full-text articles were obtained using the predetermined selection criteria, generating 12 articles with 894 participants. A total of eight articles were finally selected for the meta-analysis. The detailed process for article selection is shown in Figure 1.

### 3.2. Study Characteristics

There were 265 male and 597 female participants with an age range of 31.80 years to 80.65 years. The range of sample size in these studies was from 36 to 125. The places of publication were mainly in mainland China (n=6, 50%) [15, 16, 21–24], three studies were published in Hong Kong [25–27], and an additional three studies were published in Canada [28], Mexico [29], and USA [30], respectively. Nine of the 12 trials were published in English-language journals, and three of them were published in Chinese-language journals. Nine of these selected articles reported using a randomized control study (RCT) design and three of them used a clinical control trial (CCT) design.  Table 1 summarizes the details of the included studies.

The duration of the NTCT varied from six to 12 weeks. Of the trials, only two used follow-up assessment at eight weeks and four weeks [30, 31]. The major intervention in selected trials was acupuncture (n=9). Seven of nine acupuncture trials mentioned that the duration of the intervention was six weeks [21–24, 26, 28, 29], and two of them reported that it was eight weeks [15, 16]. Only three studies reported using Tai Chi and Qigong as an intervention [25, 30, 31]. There were studies measuring the effects of NTCT on depressive syndromes (n=12) [15, 16, 24–26, 28–31], cortisol (n=2) [27, 29], 5-HT (n=3) [21, 24, 27], G-protein (n=1) [26], cytokine (n=3) [21, 22, 28], brain functional connectivity (n=2) [15, 16], brain activity (n=1) [25], CRP (n=1) [30], and HRV (n=1) [23]. Three trials found that in the acupuncture- and Tai Chi-medication groups, the side effects caused by medication were significantly reduced [21, 24, 30]. None of them reported any serious adverse events.

### 3.3. Quality Assessment of the Studies

As rated by the PEDro scale, the sum scores of the selected trials ranged from 5 to 8, suggesting fair-to-high study quality. It was noted that allocation concealment was not used in three-quarters of the eligible studies [15, 16, 22–24, 26–29]. More than half of the selected studies did not explicitly indicate the blinding of assessors, therapists, or participants [15, 16, 21–24, 26, 29–31] or intention-to-treatment technique [15, 16, 21, 23, 25, 28, 29]. Study quality of all eligible trials is presented in Table 2.

### 3.4. The Effects of Acupuncture

Indicators of outcome variables in the eligible studies included the Hamilton rating scale for depression (HRSD/HAMD), the Montgomery–Åsberg depression rating scale (MADRS), the Carroll rating scale for depression (CRS), and the self-rating depression scale (SDS). The most used depressive scale in these studies was the HRSD/HAMD (n=5) [22–24, 26, 28]. Two trials that estimated the effects of acupuncture on MADRS were included [15, 21].

**Figure 2:**
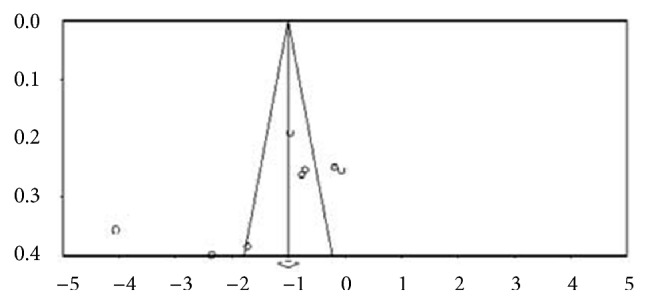
Funnel plot of publication bias for depressive syndromes.

We did not include one study in our meta-analysis because of a problem with dependent samples [16]. To detect the consistency of effects of acupuncture interventions on depressive syndromes, a sensitivity analysis was conducted by removing two trials with outlying effect sizes (*SMD *= -4.05,* SMD *= -2.35) [24, 29] based on the funnel plot and the Egger's Regression Test (intercept = -10.333,* p*=0.09) (Figure 2). After removing the outliers for further analysis, no significant difference was found using the Egger's Regression Test (intercept = -2.144,* p*= 0.67).

**Figure 3:**
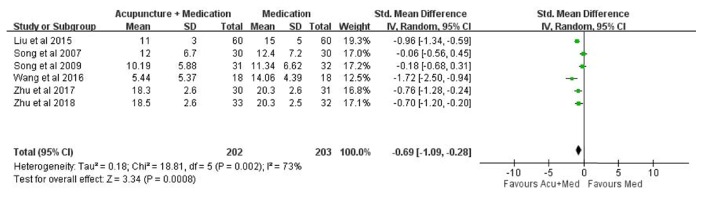
Effects of acupuncture intervention on depressive syndromes.

A total of six studies were included in this meta-analysis [15, 21–23, 26, 28]. A significant improvement was found in reducing depressive symptoms (*SMD* -0.69, 95%* CI* -1.09 to -0.28,* p*=0.002,* I*^*2*^ = 73%,* p*< 0.0008; Figure 3) with the measurement of depression scales compared to the medication group over time after the acupuncture-medication intervention.

Three trials investigated the treatment effects using the measurement of serotonin (5-HT) [21, 24, 31], and two of them showed significant benefits in the acupuncture-medication intervention group compared to the medication control group [21, 24]. A study by Song et al. [26] suggested that the intensity of the G*α* protein in depressive patients was higher than in the healthy controls, but no significant changes were found after acupuncture treatment even if the severity was considerably relieved. Vazquez et al. [29] reported a significant improvement of cortisol level, standard deviation of normal to normal R-R intervals (SDNN), and high frequency (HF) in the acupuncture-medication group, whereas a reduction of low frequency (LF) in the same group was also found [23].

Three trials reported the concentration of cytokines regarding interleukin-Iß (IL-Iß), interleukin-4 (IL-4), interleukin-10 (IL-10), interleukin-6 (IL-6), interferon-*λ* (IFN-*λ*), and tumor necrosis factor-*α* (TNF-*α*) [21, 22, 28]. Two trials suggested that IL-6 increased after six weeks of an acupuncture-medication intervention compared to the medication group [21, 22]. One trial reported an increase in concentration of TNF-*α* [28], while another study showed a contradictory result, suggesting a decreased concentration of TNF-*α* after six weeks of an acupuncture-medication intervention [22]. There was no change in IL-4 and IL-10 after the six-week intervention [21, 28]. The details of outcomes are summarized in Tables 1 and 3.

Two out of nine trials involved neuroimaging outcomes based on acupuncture techniques. Wang et al. 2017 [16] compared the changes of the resting-state functional connectivity (rsFC) in the ventral and dorsal striatal areas with the cortical cortices as well as the striatum seeds and the occipital regions between real and sham acupuncture groups. A significant increase was found after an eight-week intervention. Another study by Wang et al. 2016 [15] emphasized the increased rsFC between the left amygdala and the subgenual anterior cingulate cortex (sgACC)/pregenual anterior cingulate cortex (pgACC), and the right amygdala and left parahippocampus (Para)/putamen (Pu) (Tables 1 and 3).

### 3.5. The Effects of Tai Chi and Qigong

The indicator of depressive outcome variables from two of three trials was HRSD [27, 30], while another trial measured the change in numbers of MDD patients in each group via the BDI-II scale [25]. All the studies suggested a significant improvement in depressive syndromes in patients with MDD after Tai Chi and Qigong interventions.

Lavretsky et al. [30] showed a reduction in C reactive protein (CRP) after Tai Chi exercise compared to the health education group. Tsang et al. [31] found no statistical difference in cortisol levels after Baduanjin exercise compared to the newspaper reading group. Chan et al. [25] demonstrated that the Chan based-Dejian mind-body intervention (DMBI) significantly improved the frontal *α* asymmetry, and intra- and interhemispheric *θ* coherence in front-posterior and posterior brain regions. However, these positive findings were not found in either cognitive behavior therapy or waitlist groups.

## 4. Discussion

TCM has been practiced for over two thousand years. It is important to report the effects of NTCT and propose possible mechanisms that may help to strengthen the scientific basis of TCM. Considering the different ways of approaching diseases, we did not include studies using traditional Chinese herbs in this review. We searched empirical studies published in both English and Chinese language because, traditionally, TCM was performed in ancient China.

This is the first systematic review and meta-analysis synthesizing the effects of NTCT on MDD. We found that acupuncture may have positive effects on the treatment of MDD. This finding suggests that mixed intervention approaches may be the optimal method for the treatment of MDD. However, the evidence on whether this intervention is effective in cytokines, brain connectivity, brain structure, endocrine, HRV, and neurotransmitters is insufficient. As the three studies which mentioned their interventions were mainly based on Tai Chi and Qigong, we may not have sufficient evidence to propose plausible pathways based on mind-body exercise in the current review.

Although previous studies suggested that patients with MDD might benefit from acupuncture interventions [32, 33], the potential mechanism by which acupuncture works on MDD remains elusive. There are three plausible pathological mechanisms that may explain how the body responds to acupuncture interventions in patients with depression. First, the feedback from the hypothalamic–pituitary–adrenal axis (HPA axis) could be considered as the most important mechanism. The activity of the HPA axis is mainly related to the operation of the corticotrophin releasing hormone (CRH) from the parvocellular neurons of the paraventricular nucleus of the hypothalamus [34]. The secretion of CRH stimulates the release of the adrenocorticotropic hormone (ACTH), and the increased level of ACTH stimulates the release of glucocorticoid by the adrenal cortex, leading to an increase in concentration of cortisol. It has been widely suggested that the increased cortisol level is closely related to the severity of depressive symptoms [35–37]. Available evidence suggests that there will be a drop in the level of cortisol in patients with depression after acupuncture intervention. Thus, the dysregulation of the HPA axis can be considered as a central pathophysiological process caused by depression [38, 39].

Second, the locus coeruleus (LC) and immunity pathway would be one of the pathological reasons for depression [40]. LC is the center for synthetizing the adrenergic nerve. The ascending fibers of the adrenergic nerve are mainly projected to the amygdala, hippocampus, and limbic cortex, which are responsible for emotional changes, memory, and behavior changes. The descending fibers of the adrenergic nerve are mainly projected to the lateral dorsal horn of the spinal cord, which corresponds to the regulation of activity of the sympathetic nerve and secretion of catecholamines including epinephrine (N) and norepinephrine (NE). It has been suggested that an activated amygdala may stimulate the release of the corticotrophin-releasing hormone (CRH) that increases the activity of the sympathetic nerve via the mediating lateral dorsal horn of the spinal cord. Once the sympathetic nerve is activated, adrenaline medulla will release NE and E due to the activated adrenal gland. Thus, there is a positive bidirectional feedback loop between the CRH and the sympathetic nerve [39, 41].

Furthermore, the characteristics of the inflammatory responses are based on a complex interaction between pro- and anti-inflammatory cytokines. NE and E modulate the release of pro- and anti-inflammatory cytokines through *α*- and *β*-adrenoceptors [42]. It has been reported that the positive relationship between NE and TNF has been found, and both catecholamines of NE and E stimulated the release of IL-6 via immune cells [43–45]. When a stressful situation happens, adrenergic agents may increase due to the activation of the sympathetic nerve. This may lead to an increase of proinflammatory cytokines such as TNF, IL-1ß, and IL-6 [46]. The positive association between MDD and proinflammatory cytokines and the negative relationship between anti-inflammatory cytokines such as IL-10 and IL-4 were reported in previous studies [47–49].

Moreover, the changes of cytokines may produce behavioral changes by indoleamine 2,3-dioxygenase (IDO), which leads to a reduction of tryptophan [50]. As tryptophan is a precursor of 5-HT, the depletion of tryptophan would lead to a reduction of 5-HT. Previous efforts showing that the level of 5-HT significantly decreased in patients with depression compared to healthy controls support this finding [21]. The findings from our review are also in line with this conclusion. As the mechanism between depression and the immune system is still unclear, more studies are needed to explore underlying pathways.

**Figure 4:**
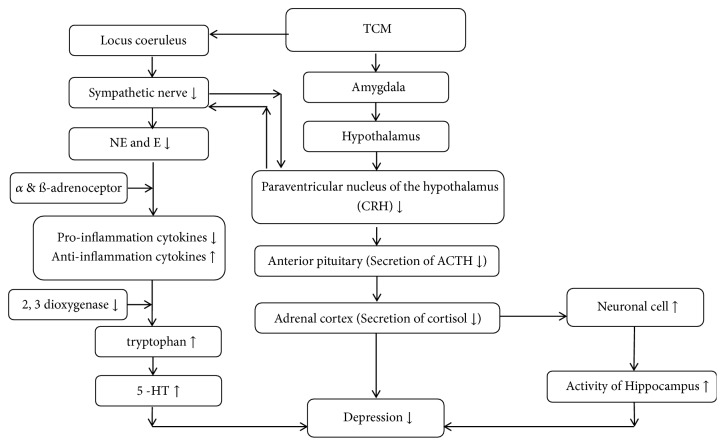
The neurological pathways associated with HPA axis, LC-immunity, and negative feedback loop of hippocampus.

Third, the negative feedback loop of the hippocampus has recently received intensive attention in studies on depression. Glucocorticoid receptors in the hippocampus are widely reported by researchers, and studies have found that the released glucocorticoid triggers a negative feedback with the hippocampus, which leads to a decreased amount of neuronal cells and eventually the hypoactivity of the hippocampus [51, 52]. Current findings with neuroimaging measurements support this possible pathway. A study by Duan et al. [53] found statistical improvement in the ratio of N-acetyl-aspartate/creatine (NAA/Cr) in the hippocampus after acupuncture interventions compared to pretreatment, which indicated that acupuncture may improve depressive syndromes by decreasing the level of cortisol and activating the activity of the hippocampus (Figure 4). Although neuroimaging studies show some beneficial effects in patients with depression after acupuncture interventions, valid conclusion cannot be drawn at this stage due to the small number of available studies. Further studies are needed to assess the changes in brain function through neuroimaging techniques.

This systematic review and meta-analysis has several limitations. First, due to great variation in neuroscience outcomes across eligible studies and the minimal number of studies in each outcome, a meta-analysis to synthesize neurological effects for MDD in the present review was not performed. Second, most of the selected studies used inadequate allocation concealment. Participants knew whether they were in the experiment or control groups even though a randomization procedure was used. Such insufficient concealment might generate subjectivity and expectation biases. Third, blinding of the intervention in many studies was absent. This might lead to an overestimation of the treatment benefits of NTCT for MDD. Fourth, because only two studies reported a follow-up period, meta-analysis to investigate the long-term effects of NTCT for MDD was not conducted. Fifth, the publication language was limited to Chinese and English. It is possible that we have missed studies published in journals of other languages.

## 5. Conclusion

In conclusion, this systematic review and meta-analysis has demonstrated that acupuncture-medication interventions produce more benefits than medication for improving depressive symptoms. The mechanisms by which acupuncture leads to positive responses for MDD might be based on the following three pathways: (1) HPA axis, (2) LC-immunity pathway, and (3) negative feedback loop of the hippocampus. Few peer-reviewed articles reported on the neurological effects of NTCT in either Chinese or English language before 2007.

Given the available NTCT options in this review, the results of traditional Chinese exercise such as Qigong and Tai Chi are inconclusive. It is difficult to summarize the current findings and propose the possible mechanisms in understanding how the body reacts when patients practice traditional Chinese exercise because the number of studies reporting neurological effects is limited. Future studies, with well-designed RCTs, should be conducted to investigate the neurological aspects of traditional Chinese mind-body exercise on MDD. Meanwhile, a cross-sectional study is needed to test the proposed plausible pathways.

## Figures and Tables

**Table 1:** Characteristics of included studies.

Source	Study design	No. of participants	Intervention group	Treatment duration	Control group	Treatment duration	Depression diagnostic criteria	Assessment
Song et al., 2007	CCT	120	N=30 Acupuncture + Fluoxetine	Acupuncture 45min/ Weekday, 6w	Con1 (n=30): fluoxetine Con2 (n=30): Sham acupuncture + placebo Con3 (n=30): No intervention (health control)	Con1: daily, 6w Con2: daily, 6w	DSM-IV	HAMD G protein in platelets G*α*s 45 G*α*i G*α*q

Tsang et al, 2013	RCT	38	N=21, Eight Section Broads	Eight Section Broads 45min/ session, 3 times/w, 12w	Con (n=17): Newspaper reading	45min/ session, 3 times/w, 12w	DSM-IV	GDS HRSD 5-HT Cortisol (salivary)

Lian et al, 2017	CCT	96	N=48 Acupuncture + Chinese herbs	Acupuncture: 4-5 times/w + Chinese herbs daily, 6w	Con (n=48): Western medicine	daily, 6w	CCMD-3	HAMD 5-HT

Lavretsky et al, 2011	RCT	73	N=36 Tai chi + Escitalopram	Tai chi 2hrs/weekly + 10mg/daily, intensity gradually increased, 10w	Con (n=37): Health education + Escitalopram	2 hrs/weekly + 10mg/daily, intensity gradually increased, 10w	DSM-IV	HRSD CRP

Chan et al, 2013	RCT	50	N=17 DMBI+ medication,	DMBI 90min/session, weekly, 10 sessions	Con1 (n=17): CBT+ medication, Con2(n=16): Medication	Con1: 90min/session, weekly, 10 sessions Con2: daily	DSM-IV	PDS via BDI-II EEG (frontal *α* asymmetry) Hemispheric *θ* coherence (inter/intra)

Song et al, 2009	CCT	125	N=31 Electro-acupuncture + placebo capsules	Acupuncture 45min/session, 3 times/w + 20mg/daily, 6 w	Con1(n=32): Fluoxetine + sham electro-acupuncture Con2 (n=32): sham electro-acupuncture + placebo capsules Con3 (n=30): No intervention (health control)	Con1: 20mg/daily +Acupuncture 45min/session, 3 times/w, 6w Con2: Acupuncture 3 times/w + 20mg/daily, 6w	DSM-IV	HRSD IL-lß TNF-a IFN-*λ* IL-4 IL-10

Vazquez et al, 2011	RCT	42	N=23 Electro-acupuncture with low frequency (4Hz)	Acupuncture 30min/session, twice/w, 6 w	Con (n=19): Sham electro-acupuncture with low frequency (4Hz) Non-therapeutic point	30min/session, twice/w, 6 w	DSM-IV	CRS SCL-90 Cortisol (salivary)

Wang et al, 2016	RCT	36	N=18 Acupuncture + fluoxetine	Acupuncture 20min/session for first 3 days, 3 days/session for the rest of 8w, +20mg/daily, 8 w	Con (n=18): Sham acupuncture + fluoxetine The same acupoints (no needle inserted)	20min/session for first 3 days, 3 days/session for the rest of 8 weeks, +20mg/daily, 8w	ICD-10	MADRS SDS fMRI rsFC

Wang et al, 2017	RCT	36	N=18 Acupuncture + fluoxetine	Acupuncture 20min/session for first 3 days, 3 days/session for the rest of 8w, +20mg/daily, 8 w	Con (n=18): Sham acupuncture + fluoxetine The same acupoints (no needle inserted)	20min/session for first 3 days, 3 days/session for the rest of 8w, +20mg/daily, 8w	ICD-10	MADRS SDS fMRI rsFC

Liu et al, 2015	RCT	120	N=60 Acupuncture + medication	Acupuncture 30min/session, alternate day, + 20-50mg, 1-2 times/daily, intensity gradually increased, 6w	Con (n=60): Medication	20-50mg, 1-2 times/daily, intensity gradually increased, 6w	ICD-10	MADRS 5-HT IL-lß IL -6 IL-4 IL-10

Zhu et al, 2018	RCT	65	N=33 Acupuncture + SSRIs	Acupuncture 30min/session, 5 times/w + daily SSRIs, 6w	Con (n=32): Western medicine	daily, 6w	CCMD-3	HAMD HRV SDNN HF LF

Zhu et al, 2017	RCT	61	N=30 Acupuncture +SSRIs	Acupuncture 30min/session, 5 times/w + daily SSRIs, 6w	Con (n=31): Western medicine	daily, 6w	CCMD-3	HAMD IL-6 TNF-*α*

Abbreviations. Con: control group; SSRI: selective serotonin reuptake inhibitors; HAMD: Hamilton depression rating scale; GDS: geriatric depression scale; HRSD: Hamilton rating scale for depression; DSM-IV: Diagnostic and Statistical Manual of Mental Disorders, 4th version; CCMD: Chinese Classification of Mental Disorders; ICD-10: the International Classification of Diseases, 10th revision; CRP: C reactive protein; 5-HT: serotonin; MADRS: Montgomery-Asberg depression rating scale; SDS: self-rating depression scale; IL: interleukin; TNF-*α*: tumor necrosis factor-*α*; IFN-*λ*: interferon-*λ*; rsFC: resting-state functional connectivity; fMRI: functional magnetic resonance imaging; DMBI: Dejian mind-body intervention; CBT: cognitive behavioral therapy; PDS: percentage of subjects reducing depressive syndrome; CRS: the Carroll rating scale; SCL-90: psychiatric symptom checklist; HRV: heart rate variability; SDNN: standard deviation of normal to normal R-R intervals; HF: high frequency; LF: low frequency.

**Table 2:** PEDro quality scale.

Source	Item 1	Item 2	Item 3	Item 4	Item 5	Item 6	Item 7	Item 8	Item 9	Item 10	PEDro
Song et al, 2007	1	0	1	0	0	1	1	1	1	1	7/10
Tsang et al, 2013	1	0	1	0	0	1	1	1	1	1	7/10
Lian et al, 2017	0	0	1	0	0	0	1	1	1	1	5/10
Lavretsky et al, 2011	1	1	1	1	0	1	0	1	1	1	8/10
Chan et al, 2013	1	1	1	1	1	1	0	0	1	1	8/10
Song et al, 2009	1	0	1	1	1	1	1	0	1	1	8/10
Vazquez et al, 2011	1	0	1	0	0	0	1	0	1	1	5/10
Wang et al, 2016	1	0	1	1	0	0	0	0	1	1	510
Wang et al, 2017	1	0	1	1	0	0	0	0	1	1	5/10
Liu et al, 2015	1	1	1	0	0	0	1	0	1	1	6/10
Zhu et al, 2018	1	0	1	0	0	1	1	0	1	1	6/10
Zhu et al, 2017	1	0	1	0	0	0	1	1	1	1	6/10

Note. Item 1: randomization; Item 2: concealed allocation; Item 3: similar baseline; Item 4: blinding of participants; Item 5: blinding of therapists; Item 6: blinding of assessors; Item 7: key outcome measures from more than 85% of subjects; Item 8: intention-to-treat; Item 9: between group difference; Item 10: point measures and measures of variability; 1: explicitly described and present in details; 0: absent, inadequately described, or unclear.

**Table 3:** Summary of neurophysiological outcomes.

	5-HT	G*α* protein	Cortisol	C reactive protein	TNF-*α*	IL-lß	IFN-*λ*	IL-4	IL-10	IL -6	EEG	Neuroimaging outcome	HRV
Song et al., 2007		v											
Tsang et al, 2013	v		v										
Lian et al, 2017	v												
Lavretsky et al, 2011				v									
Chan et al, 2013											v		
Song et al, 2009					v	v	v	v	v				
Vazquez et al, 2011			v										
Wang et al, 2016												v	
Wang et al, 2017												v	
Liu et al, 2015	v					v		v	v	v			
Zhu et al, 2018													v
Zhu et al, 2017					v					v			

## Appendix

### Search Terms

  #1 depression
  #2 major depressive disorder
  #3 depressed mood
  #4 mood disorder
  #5 affective disorder
  #6 dysthymia
  #7 #1 OR #2 OR #3 OR #4 OR #5 OR #6
  #8 neuroscience
  #9 EEG
  #10 blood sample
  #11 endocrine
  #12 neurological pathway
  #13 neuroanatom*∗*
  #14 neuroimag*∗*
  #15 neurotransmitter
  #16 scientific basis
  #17 #8 OR #9 OR #10 OR #11 OR #12 OR #13 OR #14 OR #15 OR #16
  #18 massage
  #19 complementary therapies
  #20 musculoskeletal manipulations
  #21 traditional medicine
  #22 East Asian traditional medicine
  #23 acupuncture
  #24 Qigong
  #25 mind-body therapies
  #26 breathing exercises
  #27 Tai Chi
  #28 Baduanjin
  #29 #18 OR #19 OR #20 OR #21 OR #22 OR #23 OR #24 OR #25 OR #26 OR #27 OR #28
  #30 adults
  #31 human
  #32 #30 OR #31
  #33 #7 AND #17 AND #29 AND #32
